# Causes and Treatment of Functional Dyspepsia

**Published:** 1910-05

**Authors:** 


### Causes and Treatment of Functional Dyspepsia.

Drummond, in *The British Medical Journal,* states that while it is a matter of scientific interest to diagnose accurately the special form of indigestion with which one has to deal, it is of greater practical importance to discover the cause and to point the direction in which relief may be obtained. The investigations of the secretory activity of the stomach, followed by the prescription of an appropriate diet and acid or antacid remedies, are valuable in many cases for giving relief, but are only too often insufficient for a cure. The great majority of cases of functional dyspepsia are due to obvious causes, errors of diet, bolting of food, dental deficiency and imperfect mastication, irregularity of meals, chronic bacterial intoxication, or they may be secondary to Bright's disease, phthisis, or other chronic debilitating disease, but a few of the causes are apt to be elusive. The subjects of excessive eating or drinking are apt to be reticent and much may depend upon the ability of the physician to secure their confidence and to learn their personal habits. The patient may be indiscrete in the quality of his diet as well as in the quantity. In the matter of special diet there was a great temptation for the physician to skate on thin ice of quackery and to insist upon the most minute details. It was well within the mark to state that some of the most distinguished physicians had lowered both themselves and the profession by undue exactitude in the matter of dietetic details. It was most undesirable to encourage any patient to dwell anxiously on the details of diet. In ordinary cases it was well to keep along broad lines of information and advice. There is a class of patients in whom mental disturbance produced dyspepsia. These were to be distinguished from those commonly known as dyspeptic neurotics. It is common for both the doctor and the patient to attribute depression to the dyspepsia, when the dyspepsia is really induced by the depressed state of mind brought about by other conditions. If the environment or life circumstance that is responsible for the depression can be removed the dyspepsia is soon cured. If the environment can not be changed, the patient may be encouraged to take a different attitude toward the disturbing circumstance, or a brighter view of life. One of the most useful therapeutic agents in stomach cases is rest in bed. This measure is often effective whether the excessive exertion has been physical or mental. A number of neurasthenic cases will only be benefited by rest and isolation from home influences.—*Charlotte Medical Journal.*

